# 4H leukodystrophy caused by a homozygous *POLR3B* mutation: Further delineation of the phenotype

**DOI:** 10.1002/ajmg.a.61600

**Published:** 2020-04-22

**Authors:** Eline A. Verberne, Lotje Dalen Meurs, Nicole I. Wolf, Mieke M. van Haelst

**Affiliations:** ^1^ Department of Clinical Genetics, Amsterdam UMC University of Amsterdam Amsterdam Netherlands; ^2^ Department of Pediatrics Fundashon Mariadal Kralendijk Bonaire Netherlands; ^3^ Department of Child Neurology, Emma Children's Hospital, Amsterdam UMC, and Amsterdam Neuroscience Vrije Universiteit Amsterdam Netherlands; ^4^ Department of Pediatrics Meander Medical Centre Amersfoort Netherlands

**Keywords:** 4H leukodystrophy, *POLR3B* gene, hypomyelination, hypodontia, hypogonadotropic hypogonadism

## Abstract

4H leukodystrophy, also known as Pol III‐related leukodystrophy, is a rare autosomal recessive neurodegenerative disorder characterized by hypomyelination, hypodontia, and hypogonadotropic hypogonadism. It is caused by biallelic mutations in *POLR3A*, *POL3RB*, or *POLR1C.* So far, only two patients have been described with homozygosity for the common c.1568T>A (p.Val523Glu) *POLR3B* mutation, both of them showing a remarkably mild clinical course. Here, we report another patient with homozygosity for the same mutation, but with a more severe phenotype including ataxia, developmental delay, and intellectual disability. This information is of importance for clinicians to provide comprehensive counseling to patients with 4H leukodystrophy and their families.

## 1 INTRODUCTION

4H leukodystrophy, also known as Pol III‐related leukodystrophy, is a rare autosomal recessive neurodegenerative disorder characterized by hypomyelination, hypodontia, and hypogonadotropic hypogonadism. Age of onset is usually early childhood with a progression of motor dysfunction due to increasing ataxia (Wolf et al., 2014). Other features include cognitive impairment, short stature, and myopia. The clinical course of 4H leukodystrophy is highly variable, with some patients never being able to walk independently and having mild to moderate intellectual disability, while other reported cases present only in adolescence with idiopathic hypogonadotropic hypogonadism (Richards et al., 2017; Wolf et al., 2014).

4H leukodystrophy is caused by mutations in *POLR3A*, *POL3RB*, or *POLR1C* (Bernard et al., 2011; Saitsu et al., 2011; Tetreault et al., 2011; Thiffault et al., 2015). *POLR3A* and *POLR3B* encode the largest and second largest subunits (RPC1 and RPC2, respectively) of RNA polymerase III (Pol III). Together, RPC1 and RPC2 form the catalytic center of Pol III. Pol III is an enzyme involved in the transcription of small noncoding RNAs (such as tRNAs, 5S RNA, 7SK RNA, and U6 RNA) that play a role in processes such as transcription regulation, RNA processing, ribosome assembly, and translation, which ultimately lead to protein synthesis. The transcription of small noncoding RNAs by Pol III plays an essential role in cell growth and differentiation (Dumay‐Odelot, Durrieu‐Gaillard, Da Silva, Roeder, & Teichmann, 2010). Recently, it was discovered that 4H leukodystrophy can also be caused by biallelic pathogenic variants in *POLR1C*, another subunit of Pol III (Thiffault et al., 2015). It is hypothesized that mutations in *POLR3A*, *POLR3B*, or *POLR1C* lead to a dysregulation of Pol III and thus to inadequate levels of certain tRNAs, which are needed for the synthesis of proteins essential for central nervous system myelination (Bernard et al., 2011; Saitsu et al., 2011; Thiffault et al., 2015).

The most commonly encountered *POLR3B* mutation in 4H leukodystrophy is c.1568T>A (p.Val523Glu). The majority of patients are compound heterozygous and carry a second (different) mutation in addition to c.1568T>A. Only two patients have thus far been reported with homozygosity for this mutation, both of them showing a remarkably mild clinical course.(Wolf et al., 2014)

Here, we describe a third patient with 4H leukodystrophy due to homozygous c.1568T>A (p.Val523Glu) mutations in *POLR3B*. Our patient presents with ataxia, intellectual disability, developmental delay, hypogonadotropic hypogonadism, myopia, hypodontia, and short stature, demonstrating that this genotype can also result in a more severe phenotype. This information is of importance for clinicians to provide comprehensive counseling including prenatal options to family members of patients with 4H leukodystrophy. Written informed consent for publication was obtained from the mother of the patient.

## 2 CASE REPORT

The proband, a 21‐year‐old woman, was the first child of healthy non‐consanguineous parents of Dutch Caribbean ancestry. She was born at term after an uncomplicated pregnancy and delivery, with a birth weight of 3.5 kg. At the age of 1½ years parents noticed a delay in her development, as she was not able to walk without support. When she was 2 years old, she was evaluated by a pediatrician and a neurologist. Laboratory evaluation (blood cell count, electrolytes, renal function, liver enzymes, cholesterol, thyroid‐stimulating hormone, free T4) showed no abnormalities. A computer tomography scan of the brain was performed and showed a wide fourth ventricle with a dilated cisterna magna and hypoplasia of the cerebellar vermis, which was interpreted as a Dandy‐Walker variant. She was diagnosed with infantile encephalopathy with ataxia. Since there was no permanent pediatric care on the island at that time, no follow‐up took place.

**FIGURE 1:**
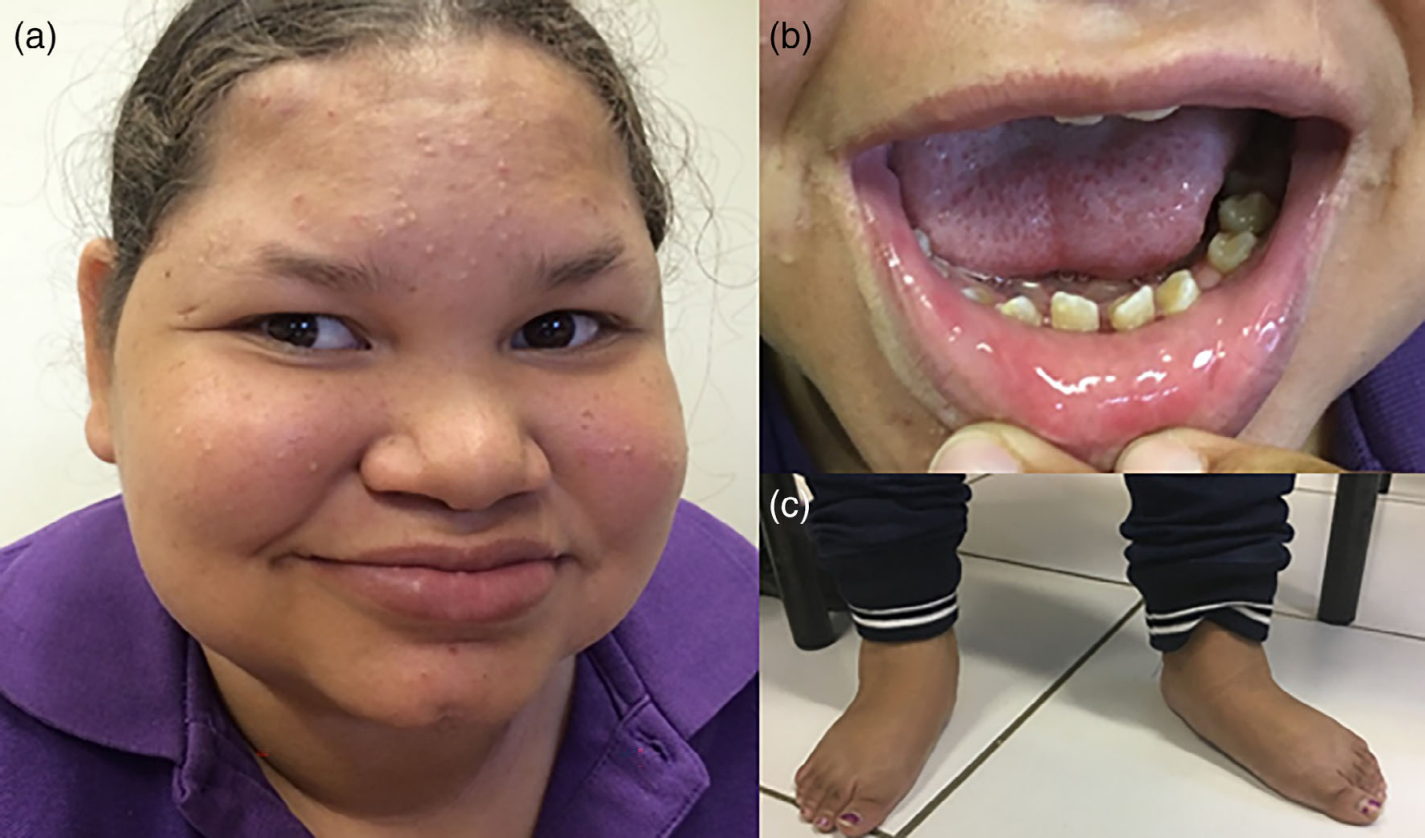
Patient at the age of 16 years. Dysmorphic features include short philtrum, thick everted lower lip, and lateral flaring of the eyebrows (a), hypodontia (b), and pes planus of both feet (c) [Color figure can be viewed at wileyonlinelibrary.com]

At the age of 14 years, she presented at the pediatric genetic clinic because her parents wanted to know the cause for her developmental delay. At that time, she had two healthy younger brothers. She used a walker because of ataxia. She could only produce three‐word sentences and there was dysarthria. Her IQ was estimated to be 40. On examination, her height was 143 cm (< ‐2 SD), weight was 66 kg (+4 *SD*), and head circumference was 54 cm (−0.5 *SD*). She was noted to have a short philtrum, thick everted lower lip, lateral flaring of the eyebrows, hypodontia, and pes planus (Figure 1). There was cerebellar ataxia with problematic gait balance and an intention tremor. A gaze‐evoked nystagmus was observed. She had bilateral myopia (−3.50/−5.50 dpt). Fundus examination revealed no abnormalities. Upon examination at the age of 15 years, she had normal secondary sex characteristics (Tanner stage M4P4) but she did not yet have her menarche. Her plasma level of luteinizing hormone (LH) was 0.5 IU/L, and the level of follicle‐stimulating hormone (FSH) was 2.5 IU/L. With a luteinizing hormone‐releasing hormone (LHRH) stimulation test, there was no significant LH or FSH response. Abdominal ultrasound showed no abnormalities.

**FIGURE 2:**
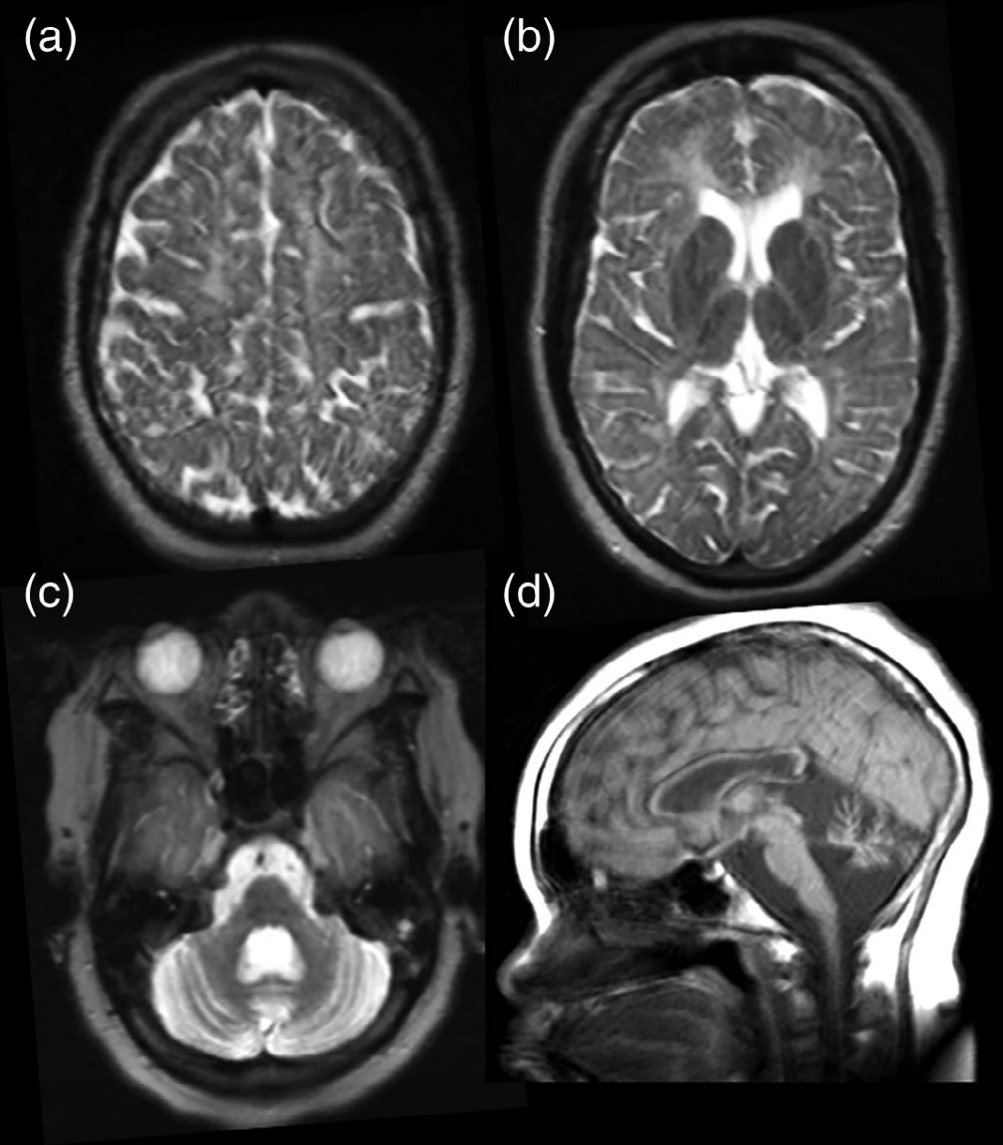
This brain MRI was performed at the age of 17 years. (a–c) are T2‐weighted axial images, demonstrating diffuse hyperintense signal of the white matter, mild supratentorial atrophy, and severe cerebellar atrophy. The ventrolateral thalamus (b) and the medial lemniscus (c) are relatively hypointense, as often seen in 4H leukodystrophy. The sagittal T1‐weighted image (d) demonstrates cerebellar atrophy and a thin corpus callosum

Single nucleotide polymorphism (SNP) array showed a normal female profile with several large regions of homozygosity. Gene panel analysis of 761 genes associated with intellectual disability (virtual panel by whole exome analysis) revealed a homozygous pathogenic missense mutation in *POLR3B*, c.1568T>A p.(Val523Glu), establishing the diagnosis of 4H leukodystrophy. Both parents were carriers. In retrospect, one of the regions of homozygosity in the proband comprises the *POLR3B* gene. After this diagnosis a brain magnetic resonance imaging (MRI) was performed, which showed features consistent with 4H leukodystrophy (Figure 2).

At the age of 20 years, she was referred to the ophthalmologist because of a white glaze on her left pupil. She was diagnosed with mature cataract of the left lens for which subsequently a cataract extraction was performed. A year later cataract of the right lens was diagnosed, for which an operation is planned.

## 3 DISCUSSION

The c.1568T>A substitution is the most commonly described *POLR3B* mutation in 4H leukodystrophy and is reported in the Genome Aggregation Database (gnomAD) with an allele frequency of 0.0003% (https://gnomad.broadinstitute.org/). Almost all of these controls were from European descent and there were zero homozygotes. Daoud et al. showed that carriers of this mutation share a common haplotype, suggesting that this mutation derives from a single ancestor (Daoud et al., 2013). Given the history of Spanish and Dutch colonization of the Caribbean island our patient was born, it could very well be that her parents have a shared European ancestor from which the mutation was inherited. In support of this, array analysis showed a region of homozygosity overlapping the *POLR3B* gene in our patient.

Homozygosity for this pathogenic variant was thus far reported in only two patients (a sibling pair) with 4H leukodystrophy. They were both mildly affected, with the older sister having no clinical symptoms of 4H leukodystrophy other than myopia at the age of 26 years. The younger brother was diagnosed with a learning disability at the age of 11 years and was referred to the neurology clinic at age 15 because of a tonic‐clonic seizure. Neurological examination showed myopia and some stumbling on tandem gait testing. One year later, he had abnormal upgaze saccades, hyperreflexia, and mild dysmetria on examination. At the age of 23 years, he did not have any new neurological deficits. Their brain MRIs showed diffuse hypomyelination with relative preservation of specific structures and significantly more residual myelin than typically seen in 4H leukodystrophy (DeGasperis, Bernard, Wolf, Miller, & Pohl, 2020).

This is the first report showing that homozygosity for the c.1568T>A *POLR3B* mutation can have a typical 4H phenotype as well. Symptoms in our patient already started in early childhood with delayed motor development. She later developed cerebellar signs including nystagmus, intention tremor, and ataxia, for which the use of a walker was required and was found to have a severe intellectual disability. Other characteristic clinical features of 4H leukodystrophy are present as well, that is, hypodontia, hypogonadotropic hypogonadism, short stature, and myopia. There is no clinical suspicion of an additional syndrome causing her severe symptoms, as all clinical, radiologic, and genetic features in our case are consistent with 4H leukodystrophy. Also, array results were normal and no other pathogenic variants were detected by intellectual disability gene panel analysis. It is known that the severity of 4H leukodystrophy can be highly variable, even within the same family, which is in line with our finding (Bernard et al., 2011; Wolf et al., 2014).

Additionally, it is of interest that our patient developed cataract as an adolescent. In a cohort of 105 mutation‐proven cases of 4H leukodystrophy, cataract was present in only three patients, including one sibling pair (Wolf et al., 2014). Furthermore, three other cases of cataract in 4H leukodystrophy have been reported (Jurkiewicz et al., 2017; Sato et al., 2011). This additional case suggests that cataract is indeed a feature of 4H leukodystrophy, although its manifestation seems to be infrequent.

In conclusion, we demonstrate that homozygosity for the common c.1568T>A (p.Val523Glu) *POLR3B* mutation causing 4H leukodystrophy can have a severe clinical phenotype. This information is important for clinicians to provide adequate (prenatal) counseling of parents of patients with this genotype.

## CONFLICT OF INTEREST

The authors have no conflicts of interest to disclose.

## AUTHOR CONTRIBUTIONS

Eline Verberne contributed to the concept, design and manuscript writing, under supervision of Mieke van Haelst. Lotje Dalen Meurs collected and interpreted patient data together with Mieke van Haelst and contributed to the manuscript writing. Nicole Wolf contributed to the interpretation of the patient data and helped supervising the project. All authors commented on the draft and approved the final manuscript.

## Data Availability

Data sharing is not applicable to this article as no new data were created or analyzed in this study.
